# Unraveling the Morphological Evolution and Etching Kinetics of Porous Silicon Nanowires During Metal-Assisted Chemical Etching

**DOI:** 10.1186/s11671-017-2156-z

**Published:** 2017-06-02

**Authors:** Lester U. Vinzons, Lei Shu, SenPo Yip, Chun-Yuen Wong, Leanne L. H. Chan, Johnny C. Ho

**Affiliations:** 1 Department of Electronic Engineering, City University of Hong Kong, Kowloon, Hong Kong; 2 Department of Physics and Materials Science, City University of Hong Kong, Kowloon, Hong Kong; 3 Shenzhen Research Institute, City University of Hong Kong, Shenzhen 518057, People’s Republic of China; 4 Department of Biology and Chemistry, City University of Hong Kong, Kowloon, Hong Kong; 5 Center for Biosystems, Neuroscience, and Nanotechnology, City University of Hong Kong, Kowloon, Hong Kong; 6 State Key Laboratory of Millimeter Waves, City University of Hong Kong, Kowloon, Hong Kong

**Keywords:** Silicon nanowire, Metal-assisted chemical etching, Silver catalyst, Silicon nanostructure, Porous silicon

## Abstract

Many potential applications of porous silicon nanowires (SiNWs) fabricated with metal-assisted chemical etching are highly dependent on the precise control of morphology for device optimization. However, the effects of key etching parameters, such as the amount of deposited metal catalyst, HF–oxidant molar ratio (*χ*), and solvent concentration, on the morphology and etching kinetics of the SiNWs still have not been fully explored. Here, the changes in the nanostructure and etch rate of degenerately doped p-type silicon in a HF–H_2_O_2_–H_2_O etching system with electrolessly deposited silver catalyst are systematically investigated. The surface morphology is found to evolve from a microporous and cratered structure to a uniform array of SiNWs at sufficiently high *χ* values. The etch rates at the nanostructure base and tip are correlated with the primary etching induced by Ag and the secondary etching induced by metal ions and diffused holes, respectively. The H_2_O concentration also affects the *χ* window where SiNWs form and the etch rates, mainly by modulating the reactant dilution and diffusion rate. By controlling the secondary etching and reactant diffusion via *χ* and H_2_O concentration, respectively, the fabrication of highly doped SiNWs with independent control of porosity from length is successfully demonstrated, which can be potentially utilized to improve the performance of SiNW-based devices.

**Electronic supplementary material:**

The online version of this article (doi:10.1186/s11671-017-2156-z) contains supplementary material, which is available to authorized users.

## Background

Despite research breakthroughs on various novel materials, silicon remains one of the most attractive substrates for fabricating nanostructures because of its abundance in nature and the existence of well-developed techniques for device integration. In recent years, one-dimensional silicon nanostructures, such as silicon nanowires (SiNWs) and nanopillars, have continued to attract attention in a wide range of applications, such as photovoltaics [1, 2], thermoelectrics [3, 4], energy storage [5–7], flexible electronics [8], biochemical sensing [9], and biological interfacing [10]. Aside from the unique electrical, optical, thermal, and mechanical properties of such nanostructures, a main driver for their sustained appeal is the development of novel fabrication techniques that allow facile formation of the nanostructures while maintaining excellent control over morphology and physical properties. Among the various fabrication techniques, metal-assisted chemical etching (MACE) stands out from an industrial viewpoint because of its simplicity, low cost, and flexibility [11, 12]. Using MACE, wafer-scale, defect-free SiNWs with defined length, porosity, conductivity, doping level, and crystal orientation can be obtained by simply selecting the appropriate Si wafer, etchant composition, reaction temperature, and reaction time [13–23]. Control of SiNW diameter, cross section, and array pitch can also be achieved through catalyst-patterning techniques, such as nanosphere lithography [24], interference lithography [25], and block co-polymer lithography [26]. This is in contrast with other fabrication techniques, such as reactive ion etching and vapor–liquid–solid methods, which require expensive equipment and may produce nanowires with surface defects, uncontrolled crystallographic orientations, and limited cross-sectional shapes [11].

In the past decade, fabrication of SiNWs from highly doped Si wafers using MACE has been of particular interest because of the resulting porous SiNWs with high crystalline quality [14, 17, 19]. The porous structure allows the nanowires to acquire highly desirable properties, such as tunable photoluminescence [15], low thermal conductivity [27], and high specific surface area [28], making them promising materials for optoelectronics [14, 16], thermoelectrics [3, 27], photocatalysis [28, 29], and energy storage [5]. In combination with their photoluminescence and high surface area, the biocompatibility and biodegradability of porous SiNWs in physiological environments also make them suitable candidates for biolabeling [15] and drug delivery applications [30]. Although relatively porous SiNWs can be obtained with low-doped Si wafers by utilizing high oxidant concentrations in the etchant [15], the use of highly doped Si is advantageous where high electrical conductivity is necessary as it obviates the need for a post-etch doping step. This is especially true in thermoelectric applications of porous SiNWs where the boost in the figure of merit is due to the decrease in thermal conductivity without significant degradation of electrical conductivity [27]. On the other hand, it has been reported that the resistance of porous SiNWs is rather large compared to that of solid SiNWs [14], implying a tradeoff between degree of porosity and electrical conductivity.

In order to realize the potential of porous SiNWs in the abovementioned applications, it is imperative to fully understand the effects of various etching parameters during MACE of highly doped Si. A number of studies [13–17, 19–21, 23] have successfully fabricated highly doped SiNWs with different lengths and porosities using MACE in HF–H_2_O_2_ etchant. Their investigations have shed light on the effect of H_2_O_2_ concentration [14–17, 20, 21, 23], HF concentration [21], HF–H_2_O_2_ volume ratio [19], etch duration [14, 16, 17, 19–21, 23], and etch temperature [19–21, 23] on the porosity [14, 16, 17, 19, 20], length [16, 17, 19–21, 23], etch rate [15], and overall morphology [15, 16, 21] of the SiNW arrays. Nevertheless, systematic studies on the effect of etchant HF–H_2_O_2_ molar ratio, defined as *χ* = [HF]/([HF] + [H_2_O_2_]), and H_2_O concentration on the formation of highly doped Si nanostructures are limited. This is despite the fact that *χ* is a key parameter in determining the morphology and etch rate of Si nanostructures [18, 31], while [H_2_O] is pivotal in the formation of SiNWs in micro-patterned areas [18]. To date, only Chiappini et al. [15] and Balasundaram et al. [19] have utilized a wide range of *χ* values (0.4–0.98 and 0.7–0.99, respectively) in the fabrication of highly doped SiNWs, while none have fully explored the effect of etchant [H_2_O]. Furthermore, despite a number of studies elucidating the mechanism for the morphological evolution, porosification, and tapering of both lightly and highly doped SiNWs fabricated with MACE [15, 17, 32], the mechanism of length evolution due to the competing effects of deposited metal etching, re-nucleated metal etching, hole diffusion, and reactant diffusion has not been fully investigated. In this study, two-step MACE [33] on degenerately doped p-type Si wafers using electrolessly deposited Ag catalyst and H_2_O_2_ oxidant was performed. Compared with MACE using patterned Au catalyst, electroless deposition can form only randomly distributed SiNWs with varied diameters [13], while Ag may limit the achievable SiNW aspect ratios due to its faster dissolution than Au [11]. Nevertheless, the use of electroless deposition and Ag catalyst in MACE is considered to be the simplest and cheapest way of forming Si nanostructures, including SiNWs. In order to explore the effects of etchant composition on the resulting nanostructures and etch rates, etchants with a relatively wide range of *χ* and [H_2_O] values were utilized. By determining the etching kinetics at the tip and base of the Si nanostructures, insight on the effects of hole injection and Si dissolution rates, secondary etching induced by dissolved metal ions and diffused holes, and diffusion rate of reactants on the resulting morphology and length of the nanostructures is provided. Highly doped SiNWs of the same lengths were also fabricated using etchants composed of different *χ* and [H_2_O] values, thereby showing that porosity can be effectively tuned by etchant composition despite varying etching durations.

## Methods

Single-side polished boron-doped p-type Si (100) wafers with a resistivity of 0.001–0.005 Ω cm and a 50-nm thermal oxide layer on the polished side were used as the starting material. The wafers were thoroughly cleaned with deionized (DI) water, acetone, and ethanol, and the thermal oxide was stripped off with dilute HF, resulting in H-terminated Si surfaces. To confine etching on the polished Si surface, the backside of the Si wafers were coated with photoresist (AZ5214). Si samples for the MACE experiments were obtained by cleaving the wafers into 1 × 1 cm^2^ pieces. Si samples from the same wafer were used for each set of experiments.

**Fig. 1:**
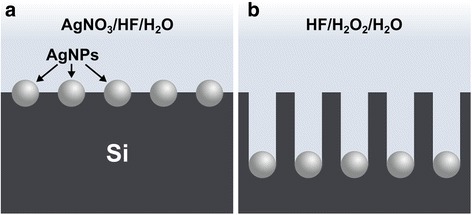
Schematic cross-sectional view of the fabrication of Si nanostructures using two-step MACE. **a** In the first step, AgNPs are electrolessly deposited on the Si surface in an aqueous solution containing AgNO_3_ and HF. **b** In the second step, the AgNPs catalyze the etching of the Si substrate in a solution composed of HF, H_2_O_2_, and H_2_O, leading to the formation of Si nanostructures

MACE was carried out using a two-step etching process consisting of electroless deposition of Ag catalyst and then etching in HF–H_2_O_2_ solutions, as shown schematically in Fig. 1. The Si pieces were first immersed in 5% HF for 3 min. Electroless deposition of Ag nanoparticles (AgNPs) on the Si substrate was then performed in an aqueous solution containing 4.8 M HF and 0.005 M AgNO_3_ for different time durations (Fig. 1a). After mild rinsing with DI water, the AgNP-coated Si samples were etched in aqueous HF–H_2_O_2_ solutions with different *χ* and [H_2_O] values (see Additional file 1: Table S1) for different lengths of time (Fig. 1b). The etched Si samples were thoroughly rinsed with DI water and then immersed in 1:1 (*v*/*v*) HNO_3_ for 10 min to dissolve the AgNPs. After another thorough DI water rinse, the Si samples were soaked in 5% HF for 3 min to remove any formed oxide layer and then rinsed again with DI water multiple times. Ag deposition and HF–H_2_O_2_ etching were performed in the dark. All samples were processed at room temperature (22–23 °C) in separate plastic beakers containing 15 ml of the required solution.

The etched Si surfaces were characterized by scanning electron microscopy (SEM, Phenom Pro or FEI/Philips XL-30) and transmission electron microscopy (TEM, Philips CM20). To prepare the samples for SEM observation, the photoresist at the backside was removed by acetone. The Si samples were then rinsed with ethanol and dried on a hotplate to minimize SiNW agglomeration due to water evaporation [19].

## Results and Discussion

### Silver Catalyst Deposition and Formation of Porous SiNWs

**Fig. 2:**
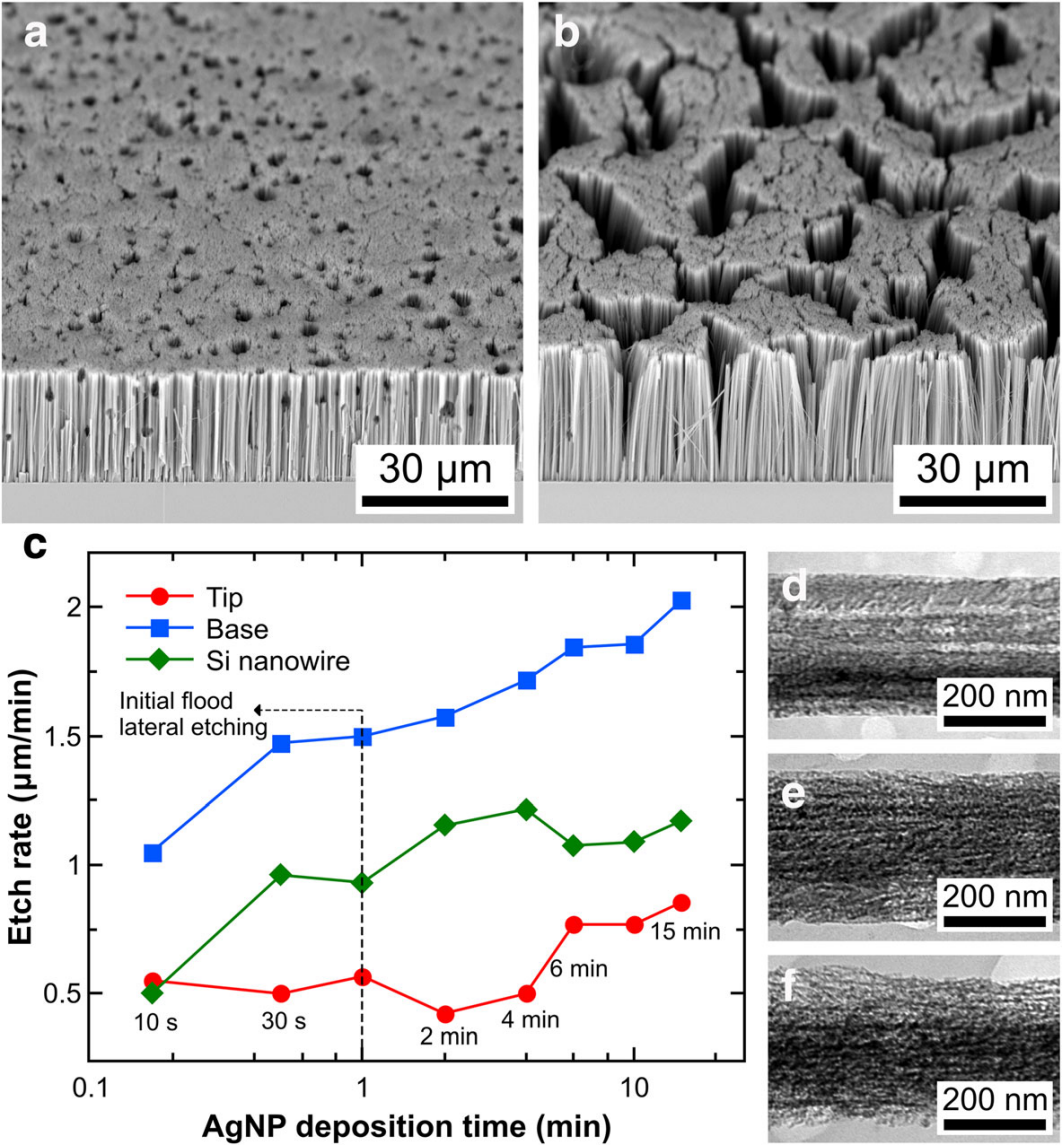
Fabricated SiNWs using a Ag deposition solution containing 0.005 M AgNO_3_ and 4.8 M HF and an etchant solution composed of 48 M H_2_O and 0.95 HF–H_2_O_2_ molar ratio. SEM images of the SiNWs for Ag deposition times of **a** 30 s and **b** 4 min. **c** Etch rate of the SiNWs for different Ag deposition times. TEM images of the middle section of the SiNWs for Ag deposition times of **d** 4, **e** 10, and **f** 15 showing the degree of porosity. Etch duration for all samples was 30 min

The effect of Ag deposition duration (10 s to 15 min) in a solution of 0.005 M AgNO_3_ and 4.8 M HF on the formation of highly doped SiNWs was investigated. When the Ag-loaded samples were etched in a solution containing 4.8 M H_2_O and *χ* = 0.95 for 30 min, short deposition times (≤2 min) resulted in a dense array of vertical pores with some lateral pits, as shown in Fig. 2a. However, the pores were not dense enough to form well-separated nanowire structures if the deposition time is ≤1 min. On the other hand, SiNWs which had almost no defects were obtained with a Ag deposition time of 4 min (Fig. 2b), with higher deposition times resulting in pit-free SiNWs. Aside from changes in the SiNW morphology, a non-monotonic variation in SiNW etch rate with respect to Ag deposition time was also observed, as shown in Fig. 2c (diamond symbols). The SiNW etch rate increased from a deposition time of 10 s to 4 min but decreased unexpectedly between 4 min and 6 min. Afterwards, the etch rate increased again until 15 min, albeit with relatively lower etch rate values.

In order to account for the peculiar trend in the SiNW etch rate, the etch rates at the tip and base of the SiNWs were also determined from the cross-sectional SEM images by aligning the micrograph of an etched Si sample with that of an unetched Si sample from the same wafer at the backside (see Additional file 1: Figure S1). From Fig. 2c, it can be seen that the etch rate at the SiNW base (square symbols) monotonically increases with AgNP deposition time. On the other hand, three domains can be seen from the trend line of the SiNW tip etch rate (circle symbols): (a) ≤1 min, where the etch rate is relatively high; (b) between 1 and 6 min, where the etch rate is relatively low; and (c) ≥6 min, where the etch rate is the highest. Thus, the initial increase in SiNW etch rate from the deposition time of 10 s to 1 min was due to the constant increase in etch rate at the base, while the subsequent increase in etch rate at deposition times of 2 and 4 min was due to the relatively low etch rates at the tips. Meanwhile, the drop in SiNW etch rate at the deposition time of 6 min and the relatively low etch rates in the succeeding deposition times were caused by the increase in etch rate at the tips.

Since the surface coverage of AgNPs is directly related to the Ag deposition time [22, 34, 35], the lateral pitting for low deposition times can be attributed to a sparse Ag network [18, 34, 36] with some isolated AgNPs moving in random <100> directions due to their irregular shape [37]. The SiNW etch rate at the base is expected to increase with Ag deposition time due to the increase in the amount of Ag catalyst, which provides more surface area for hole injection by H_2_O_2_. Moreover, a higher amount of Ag also means more oxidized Ag by H_2_O_2_ [15, 17], resulting in a higher concentration of Ag^+^ ions that can contribute to Si dissolution via a galvanic displacement reaction [31]. On the other hand, the relatively high etch rates at the tips for Ag deposition times ≤1 min can be explained by an initial flood lateral etching caused by irregularly shaped AgNPs from the sparse Ag network. The subsequent increases in tip etch rate with increasing Ag deposition time can be attributed to increasing rates of metal re-nucleation [17, 38] at the tips and, to a lesser extent, hole diffusion [31, 39].

There was an overall increase in the porosity of the fabricated SiNWs as the Ag deposition time increased, as shown in Fig. 2d–f. (See Additional file 1: Figure S2a–c for supplementary TEM images.) Such increases in porosification are expected due to higher concentrations of Ag^+^ ions, which result in re-nucleated metal and ion-induced etching [15, 17, 38]. Furthermore, higher rates of hole injection might have also resulted in more diffused holes, which can contribute to pore formation [19, 39]. The porous structure of the SiNWs appears to occur as a porous shell in most nanowires, similar to what was observed in previous studies [14, 17]. For some SiNWs with a Ag deposition time of 15 min, the sidewalls appear considerably rougher with relatively large (≈20-nm diameter) dark spots, probably due to larger re-nucleated Ag particles. However, the porosity distribution is not clear-cut: some SiNWs with a Ag deposition time of 4 min have almost the same porosity as that of SiNWs with a Ag deposition time of 10 min; the same can be said of SiNWs with Ag deposition times of 10 and 15 min. This was probably due to SiNWs obtained from different areas of the sample, which experienced slightly different porosification rates depending on the actual amount of deposited (or re-nucleated) AgNPs and the concentration of unreacted etchant, which is expected to be higher towards the sides of the sample. Nevertheless, the general trend observed here indicates that the overall amount of deposited Ag provides another degree of freedom in controlling the porosity of SiNWs.

The degree of porosity increases from the base to the tip of the SiNWs (see Additional file 1: Figure S2d–l), similar to that in previous studies [16, 19]. This is expected from the longer exposure of the upper regions of the nanowires to the etchant [19]. On the other hand, majority of the SiNWs have a tapered longitudinal profile, which is characteristic of highly doped SiNWs fabricated using Ag-MACE due to the continuous dissolution of the AgNPs at the SiNW base and re-nucleation on other sites [15, 17]. Nevertheless, there were a few SiNWs having a slightly biconic or hourglass profile, i.e., the middle cross-section is either larger or smaller, respectively, than both the top and bottom cross sections. This suggests that the mobile Ag^+^ ions could be redepositing not only onto upper sections of nanowires but also onto neighboring AgNPs at the base.

### Etchant Composition and Morphological Evolution of Si Nanostructures

**Fig. 3:**
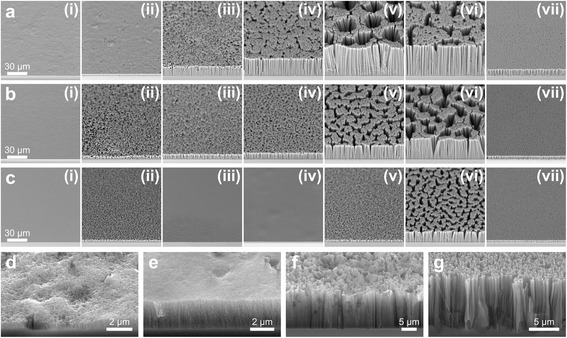
SEM images of the Si surface etched in solutions with H_2_O concentrations of **a** 46 M, **b** 48 M, and **c** 50 M and HF–H_2_O_2_ molar ratios of *(i)* 0.7, *(ii)* 0.75, *(iii)* 0.8, *(iv)* 0.85, *(v)* 0.9, *(vi)* 0.95, and *(vii)* 0.99. **d**–**g** High-magnification SEM images of the samples in **a** *(i)*, *(ii*), (*iii*), and *(vii*), respectively. Samples were etched in HF–H_2_O_2_ for 30 min

The morphology of the resulting nanostructures in degenerately doped Si was determined using *χ* values from 0.7 to 0.99 and H_2_O concentrations of 46, 48, and 50 M. Electroless deposition of AgNPs was performed in a solution of 0.005 AgNO_3_ and 4.8 M HF for 4 min, while etching in HF–H_2_O_2_–H_2_O solutions was carried out for 30 min. Figure 3 shows the morphology of the Si surface as seen from the SEM. For [H_2_O] = 46 M and 48 M, the features on the etched Si evolves from microporous Si with craters (*χ* = 0.7 and 0.75, Fig. 3d, e), to macropores with deep pores (*χ* = 0.75 and 0.80, Fig. 3f), and then to SiNWs (*χ* ≥ 0.85). For [H_2_O] = 50 M, the evolution of the Si surface is slightly different: from polished Si (*χ* = 0.7), to macropores with deep pores (*χ* = 0.75), to microporous Si with craters (*χ* = 0.8–0.85), and then to SiNWs (*χ* ≥ 0.9). In contrast with the work of Chiappini et al. [15], SiNWs on top of a porous Si film for 0.7 < *χ* < 0.95 were not observed, and SiNW-only structures were obtained in a larger *χ* range (*χ* ≥ 0.85 instead of *χ* > 0.95). These differences were most likely due to the variations in the amount of deposited AgNPs, etching time, H_2_O concentrations, and HNO_3_ and HF post-etch treatments.

The Si morphologies obtained can be explained in terms of both current density at the Ag–Si interface and Ag^+^ ion-induced etching [15, 17, 31, 38]. The formation of SiNWs at high *χ* values (≥0.85 or 0.9) can be attributed to microporous Si formation at low current densities with subsequent dissolution of the microporous Si by polishing [31, 38]. A similar mechanism was most likely responsible for the formation of macroporous Si with deep pores at lower *χ* values, except that the higher current densities at the pore end resulted in oxide formation and subsequent hole diffusion [31], resulting in the shallow macropores at the top surface. Such hole diffusion is expected to be particularly significant for highly doped p-type Si because of the positive Schottky barrier height that pulls injected holes away from the metal–Si interface [40]. It is also possible that the relatively high [H_2_O_2_] to [HF] ratio led to significant Ag dissolution, which in turn resulted in the low density of deep pores in the Si substrate. On the other hand, the appearance of microporous Si with craters at *χ* = 0.7–0.85 indicates the occurrence of low current densities in this *χ* range, which is consistent with the observation of Chartier et al. [31] Moreover, concomitant porosification of Si by Ag^+^ ions is also expected to occur in this regime. The formation of SiNWs at a higher *χ* value for the most dilute etchant (50 M H_2_O) is consistent with the observation of Chiappini et al. [15] that higher ethanol concentrations favor the formation of porous and polished Si rather than SiNWs. These results could be due to the slow diffusion of reactants to the nanostructure base due to the low HF and H_2_O_2_ concentration gradients along the Si nanostructure depth. In this case, the impact of etching at the tips becomes relatively significant and deep pores could not be formed by the metal particles.

It should be noted that for relatively low values of *χ* (≤0.8), the morphology throughout the Si surface was not uniform (see Additional file 1: Figure S3). For [H_2_O] = 50 M, non-uniform etching also occurred for *χ* = 0.85. In all cases, a uniformly etched surface was obtained only when SiNWs were formed. For non-uniformly etched surfaces, sections with homologous morphologies tend to occur at about the same general location from the center of the sample. The non-uniform etching dynamics at different areas of the sample can be explained by the increase in the concentration of dissolved catalyst ions in the solution and their subsequent diffusion to and re-nucleation on other areas of the sample. This was facilitated to a certain extent by the concurrent outward diffusion of H_2_ bubbles as the Si was etched.

At a very high *χ* value (0.99), a very dense array of fine SiNWs was obtained, leading to a black sample surface (see Additional file 1: Figure S3a–c(vii)). However, many of these SiNWs had slanting sidewalls and some lateral pits, both of which occur near the base (Fig. 3g) and at long etching times (Additional file 1: Figure S4d). A number of bumps where nanowire formation terminated prematurely can also be seen. Such features were probably caused by the depletion of H_2_O_2_ in the etching solution that led to different rates of sinking of various portions of the AgNP film. As the Ag network disintegrated, some AgNP or small Ag film sections began etching in horizontal <100> directions.

### Etching Kinetics of Si Nanostructures During MACE

**Fig. 4:**
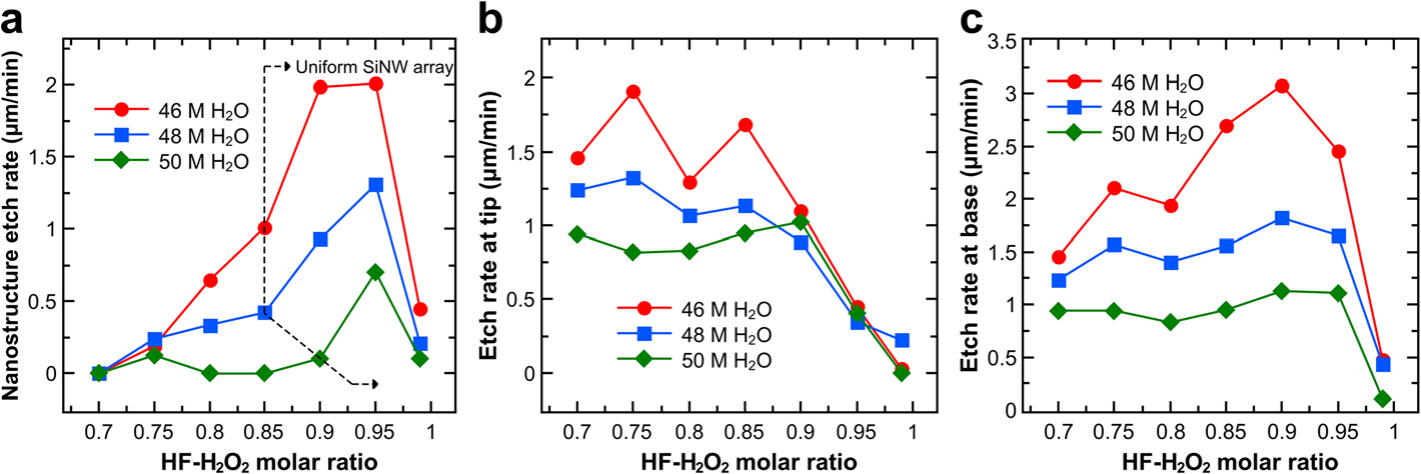
Apparent etch rates observed after 30 min of etching in solutions composed of different HF–H_2_O_2_ molar ratios and H_2_O concentrations. **a** Etch rate of the Si nanostructures based on the resulting length. **b**, **c** Etch rate of the bulk Si with respect to the tip and base of the Si nanostructures, respectively

The variation of etch rate with *χ* for different [H_2_O] values is shown in Fig. 4. The apparent etch rate of the nanostructures generally increases with *χ* and peaks at *χ* = 0.95, after which the etch rates drop significantly. This trend is true irrespective of the [H_2_O] and the morphology of the Si nanostructure. However, [H_2_O] can be seen to have considerably affected the magnitude of the etch rates, with the magnitude increasing for decreasing [H_2_O] values (i.e., more concentrated etchants). Figure 4b shows that the etch rate at the tip greatly decreased after *χ* = 0.85 for [H_2_O] = 46 and 48 M and after *χ* = 0.9 for [H_2_O] = 50 M. On the other hand, Fig. 4c shows that the etch rate at the base considerably increased at *χ* = 0.85 and 0.9 for the 46 M H_2_O etchant but only slightly for the 48 and 50 M H_2_O etchants. The etch rate at the base remained high at *χ* = 0.95 but decreased significantly at *χ* = 0.99.

From the trends in etch rates at the tip and base of the Si nanostructures, it can be determined that the increase in SiNW length for [H_2_O] = 46 M was mainly due to the acceleration of etching at the base, while the lengthening of the SiNWs for [H_2_O] = 48 and 50 M were primarily dictated by the suppression of etching at the tips. Furthermore, the trend of the overall nanostructure etch rate is not exactly the same as the trend of the etch rate at the base. In particular, the *χ* value where the peak etch rate at the base occurs and the *χ* value where the greatest nanostructure height is achieved are not the same (0.9 versus 0.95, respectively) due to the competing effect of tip etch rate.

The *χ* value where the peak SiNW length occurred (0.95) is close to that obtained by Qi et al. [21] (*χ* = 0.91) for a highly doped n-type Si substrate. The initial decrease in tip etch rate occurring near *χ* = 0.85 is consistent with the observed morphological evolution of the Si surface wherein low hole diffusion and Ag re-nucleation allowed the formation of SiNWs at *χ* ≥ 0.85. The almost concurrent drop in tip etch rate and jump in base etch rate at around *χ* = 0.85 means that holes and Ag^+^ ions that previously diffused away from the etching front at lower *χ* values were now being constrained at the base due to the inhibition of oxide formation at the Ag–Si interface. At the same time, the increased [HF] at higher *χ* values accelerated Si dissolution, leading to an increase in the Ag penetration rate. Since both [HF] and [H_2_O_2_] decrease when [H_2_O] increases for a given *χ* value (see Additional file 1: Table S1), the decrease in etch rate at the tips and base for higher [H_2_O] values is expected. The smaller increases in base etch rate between *χ* = 0.85 and 0.95 for [H_2_O] = 48 and 50 M are due to the slower diffusion of reactants through the SiNW length for more dilute etchants.

At *χ* = 0.9, the etch rate at the base is maximal due to the optimal rates of hole injection by H_2_O_2_ and Si dissolution by HF. Below this value, [HF] is the rate-determining factor as dissolution of oxidized Si is not fast enough; above this value, [H_2_O_2_] determines the reaction rate as the number of injected holes is too low. This depletion of H_2_O_2_ in the solution explains why the etch rate at the base starts to decrease at *χ* = 0.95 and steeply drops at *χ* = 0.99. Such dependency of the etch rate on both [HF] and [H_2_O_2_] was observed previously [21] and implies that both reactant concentrations should be considered in any expression for the reaction rate of MACE of Si. On the other hand, a maximum value for *χ* is not apparent from the tip etch rate. Instead, the tip etch rate is mainly determined by [H_2_O_2_]. Since etching at the tip of the Si nanostructures can be mainly attributed to metal ion re-nucleation and hole diffusion, this correlation is reasonable as higher [H_2_O_2_] values with respect to [HF] lead to greater concentrations of mobile Ag^+^ ions [15, 17] and injected holes [31, 32].

### Evolution of SiNW Length with Time for Different Etchant Compositions

The length of SiNWs is an important parameter in photovoltaic [2], energy storage [6], sensing [41], and thermoelectric applications [4]. In order to fabricate SiNWs of a specified length, the temporal variation of the SiNW length was determined for etchants with *χ* = 0.9, 0.92, 0.95, and 0.98 and [H_2_O] = 46, 48, and 50 M. For these experiments, the AgNP deposition time was increased to 10 min to minimize lateral pitting in the SiNWs fabricated with long etch times (see Additional file 1: Figure S4a–c). Likewise, the highest *χ* value utilized was 0.98 because SiNWs with slanting sidewalls and lateral pits were obtained for *χ* = 0.99 even with a AgNP deposition time of 10 min (see Additional file 1: Figure S4d).

**Fig. 5:**
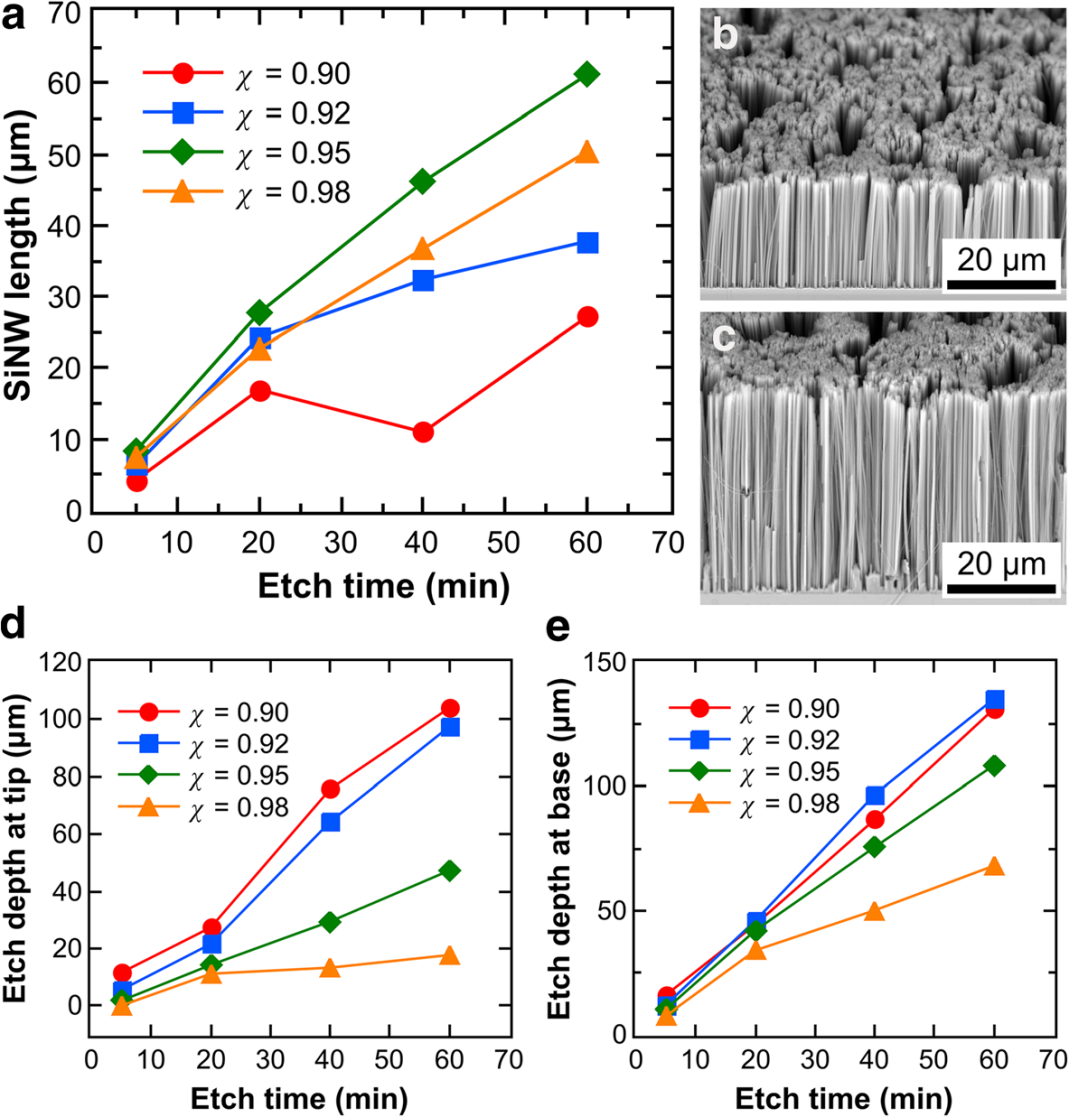
Evolution of SiNW length with time for different HF–H_2_O_2_ molar ratios at 48 M H_2_O. **a** Effect of etch time on SiNW length. **b**, **c** SEM images of SiNWs after etching for 1 h in a solution composed of 48 M H_2_O and HF–H_2_O_2_ molar ratios of 0.9 and 0.98, respectively. **d**, **e** Etched bulk Si thickness with respect to the tip and base of the SiNWs over time

Figure 5a shows that the length of the formed SiNWs increases with the MACE reaction time, consistent with observations in previous studies [14, 16–19, 21, 23]. However, it can be seen that the increase in SiNW length diminishes over time, i.e., the etch rate is decreasing (see Additional file 1: Figure S5a), which could be due to increasing etch rates at the SiNW tips or decreasing etch rates at the base or both. Figure 5d shows increasing tip etch rates over time for *χ* = 0.92 and 0.95, with the increase being more pronounced in the former (see Additional file 1: Figure S5b). This indicates an increasing amount of re-nucleated Ag at the SiNW tips, which is expected to be more significant for lower *χ* values. On the other hand, Fig. 5e shows that at etch times greater than 5 min, the etch rate at the SiNW base was almost constant for *χ* = 0.9 and 0.92 but decreasing for *χ* = 0.95 and 0.98 (see Additional file 1: Figure S5c). The latter was probably due to impeded diffusion of reactants to the SiNW base caused by the longer diffusion lengths (SiNW lengths were longer for *χ* = 0.95 and 0.98) and depletion of H_2_O_2_ in the etching solution.

It should be noted from Fig. 5 that at *χ* = 0.9, the evolution of length with time is erratic because of the non-monotonic trend of the etch rate at the tip (see Additional file 1: Figure S5b). This may be due to differing amounts of re-nucleated Ag as more mobile Ag^+^ ions were generated. Nevertheless, lower *χ* values have the advantage of forming defect-free SiNWs (Fig. 5b) as higher *χ* may result in some lateral pits, as shown in Fig. 5c for *χ* = 0.98. The trend of the overall etch rates and etch rates at the tip and base of the SiNWs with respect to *χ* mirrors those observed in Fig. 4. However, it can be seen in Fig. 5e that *χ* = 0.92, which is not included in Fig. 4, actually results in a higher base etch rate than *χ* = 0.9.

**Fig. 6:**
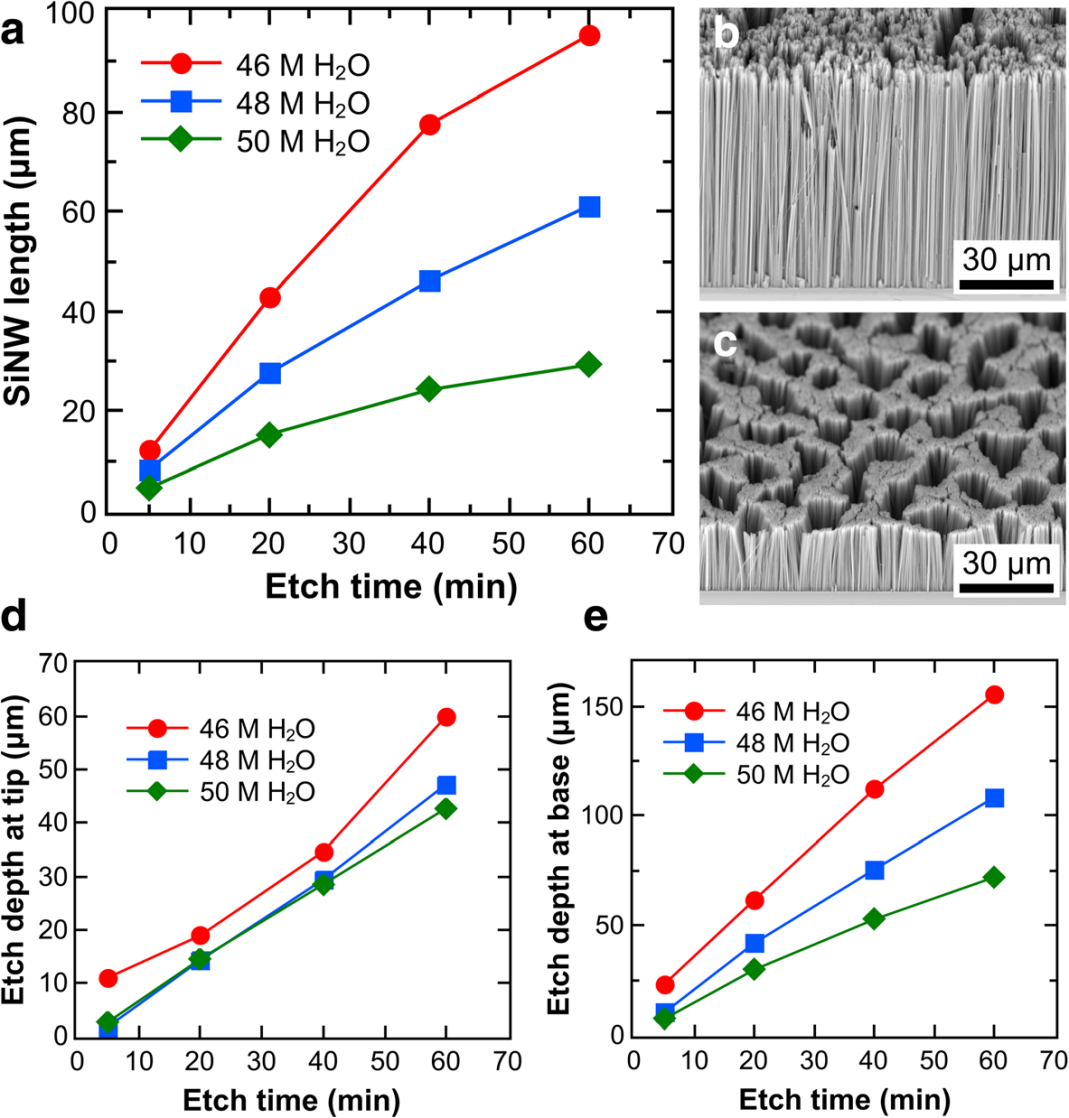
Evolution of SiNW length with time for different H_2_O concentrations at a fixed HF–H_2_O_2_ molar ratio of 0.95. **a** Effect of etch time on SiNW length. **b**, **c** SEM images of SiNWs after etching for 1 h in a solution composed of HF–H_2_O_2_ molar ratios of 0.95 and H_2_O concentrations of 46 and 50 M, respectively. **d**, **e** Etched bulk Si thickness with respect to the tip and base of the SiNWs over time

The SiNW length also increases with etch duration for more concentrated (46 M H_2_O) and dilute (50 M H_2_O) etchants, as shown in Fig. 6a. Likewise, the etch rate decreases with etch time for all H_2_O concentrations used (see Additional file 1: Figure S6a). Figure 5d, e show that, while the etch depth at the tip and base of the SiNWs both increase with time, the amount of increase at the tip is almost constant but that at the base is decreasing (see Additional file 1: Figure S6b, c). These trends are consistent with those observed for *χ* = 0.95 in Fig. 5 and Additional file 1: Figure S5. Hence, for *χ* = 0.95, the increase in the amount of re-nucleated Ag at the SiNW tips with time is negligible, while the increasing SiNW lengths progressively hampers reactant diffusion to the SiNW base. Figure 6 also shows that regardless of the etching time point, lower [H_2_O] generally results in more etched Si, similar to what was observed in Fig. 4. However, Fig. 6b, c show that low [H_2_O] solutions may result in some lateral pitting in the SiNWs whereas high [H_2_O] solutions do not. This could be due to fast etching at sites with re-nucleated Ag particles for low [H_2_O] etchants.

### Fabrication of Highly Doped SiNWs with Controlled Porosity

SiNW porosity is another key parameter in SiNW-based devices [5, 16, 27, 29], making its controlled formation during SiNW fabrication highly important. The degree of porosity of SiNWs fabricated using two-step MACE is directly related to the H_2_O_2_ concentration [14–17, 20], etching time [14, 16, 17, 20], and temperature [20] and inversely related to the HF–H_2_O_2_ volume ratio [19] and wafer resistivity [17]. In our Ag deposition experiment, SiNW porosity was also found to be directly related to the amount of deposited Ag. However, the extent of SiNW porosification cannot be easily tuned without affecting other parameters. For example, length and porosity are expected to vary together if one of them is adjusted using either [H_2_O_2_], etch time, or temperature. If a SiNW of a specific length needs to have higher porosity, the [H_2_O_2_] can be increased, but the etch duration needs to be decreased as higher [H_2_O_2_] may have a higher etch rate. This raises the question of whether the desired porosity can still be achieved given the shorter etch time.

**Fig. 7:**
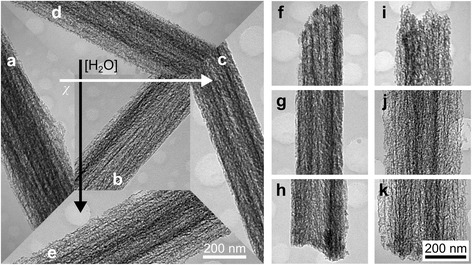
TEM images of fabricated SiNWs with a length of ≈20 μm showing the variation of porosity at the middle section with **a**–**c** HF–H_2_O_2_ molar ratio and **d**, **b**, **c** H_2_O concentration. **a**–**c** HF–H_2_O_2_ molar ratio of the etchant was 0.92, 0.95, and 0.98, respectively, with [H_2_O] = 48 M. **d**, **b**, **c** H_2_O concentration of the etchant was 46, 48, and 50 M, respectively, with HF–H_2_O_2_ molar ratio = 0.95. TEM images of SiNWs with **f**–**h** low and **i**–**k** high porosity corresponding to those shown in **c** and **e**, respectively, but including the **f**, **i** *top* and **h**, **k** *bottom* sections. The *scale bar* in **e** and **k** also applies to the images in **a**–**d** and **f**–**j**, respectively

Here, the extent of SiNW porosification caused independently by *χ* and [H_2_O] was compared for *χ* = 0.92, 0.95, and 0.98 ([H_2_O] fixed at 48 M) and [H_2_O] = 46, 48, and 50 M (*χ* fixed at 0.95). The SiNW length was fixed at 20 μm by using different etch durations based on the etch rate of the respective etchant composition (see Additional file 1: Table S2). The porosity, in general, was found to be inversely related to *χ* and directly related to [H_2_O], as shown in Fig. 7a–c and Fig. 7d, b, e, respectively. (The trends are more apparent in Additional file 1: Figure S7 where more samples are shown.) Comparing the SiNWs for *χ* = 0.95 and *χ* = 0.98 (Fig. 7b, c), the samples for *χ* = 0.95 appear to have a higher pore density and most are surrounded by a thin porous shell [14, 17]. Meanwhile, for *χ* = 0.98, some SiNWs appear to be rough and solid rather than porous, which indicates a much lesser degree of porosification. On the other hand, the overall porosity obtained for *χ* = 0.92 (Fig. 7a and Additional file 1: Figure S7a) appears to depart from the expected trend. While some SiNWs have a porous shell, others seem to be only roughened although to a higher extent compared with those for *χ* = 0.98. In contrast, the increase of porosity with [H_2_O] is more consistent. Although no rough solid SiNWs were obtained for [H_2_O] = 46 M, highly porous SiNWs were formed for [H_2_O] = 50 M unlike in the case of *χ* = 0.92.

The higher SiNW porosity obtained for *χ* = 0.95 than *χ* = 0.98 is consistent with the fact that metal re-nucleation, metal ion-induced etching, and hole diffusion are higher for lower *χ* values. It also shows that the longer etch time utilized for *χ* = 0.98 to obtain 20-μm long SiNWs did not overcome the porosification caused by the etchant *χ* value. The seemingly lesser degree of porosification for *χ* = 0.92 could not have been due to the difference in etch time as a longer etching period was utilized for it than for *χ* = 0.95. It is possible that more porous SiNWs were actually obtained for *χ* = 0.92; however, due to the very high porosity, HF–H_2_O_2_ etching of porous Si both at the surface and inside the pore walls resulted, leading to the rapid collapse of the porous shells [42] and subsequent exposure of the less porous Si surface underneath. That more porous SiNWs were obtained for higher [H_2_O] values confirms that etch duration has a significant effect on porosity [14, 16, 17, 20]. (Note the large differences in etch times in Additional file 1: Table S2 for different [H_2_O] values.) The high degree of porosity obtained for 50 M H_2_O without the collapse of the porous shell could be due to minimized HF–H_2_O_2_ etching inside the pore walls because of the lower diffusion rate of reactants in dilute etchants.

Examination of the surface roughness along the length of the SiNWs reveals the characteristic increase in porosity from the base to the tip of highly doped SiNWs fabricated with MACE [16, 19], as shown in Fig. 7f–k (also in Additional file 1: Figure S8). The increase in porosity towards the tip of the SiNWs is due to the longer exposure time of these portions in the etchant solution [19]. Figure 6f–k and Additional file 1: Figure S8 also show that the SiNWs are tapered, with the tapering being more severe in SiNWs which are more porous (Fig. 7i–k and Additional file 1: Figure S8a–c). This is expected considering that porosification is directly related to the amount of dissolution of the metal catalyst at the SiNW base [15, 17]. However, a closer look at the SiNWs reveals that some nanowires exhibit a slightly biconic or hourglass longitudinal profile, which seems to occur more often among those with higher porosity. Likewise, more porous SiNWs generally have larger diameters than the less porous ones (see Additional file 1: Figure S7). In order to achieve highly porous SiNWs with smaller diameters, it might be necessary to use a more inert catalyst like Au [32].

Based on the results obtained here, porosity control in highly doped SiNWs of a given length is possible through the use of the appropriate etchant composition. To achieve low porosity, it is best to use high *χ* values as rough solid nanowires could be obtained. To achieve highly porous SiNWs, on the other hand, the use of dilute etchants with moderate *χ* values (≈0.95) is advisable as this avoids the disintegration of the porous shell which occurs in etchants with low *χ* values. It is conceivable that lower porosities could be achieved using an etchant of both high *χ* and low [H_2_O] values and higher porosities using an etchant of both (slightly) lower *χ* and high [H_2_O] values. In the case of low-porosity SiNWs, the maximum *χ* and minimum [H_2_O] values would probably be dictated by the values where considerable lateral pitting occurs. On the other hand, for high-porosity SiNWs, the minimum *χ* and maximum [H_2_O] values would be determined by the values where the porous shell dissolves and the SiNW structures are lost, respectively. These methods can be combined with our earlier finding of porosity control via the amount deposited Ag in order to obtain a wide range of SiNW porosities.

## Conclusions

Porous SiNWs were fabricated from degenerately doped p-type Si substrates using metal-assisted chemical etching in HF–H_2_O_2_ with electrolessly deposited Ag catalyst. The effect of Ag deposition time, etchant HF–H_2_O_2_ molar ratio, and etchant H_2_O concentration on the morphology and etch rate of the Si nanostructures was systematically studied. It was shown that there is an optimal amount of deposited Ag particles necessary to form damage-free SiNWs. Furthermore, the amount of deposited Ag particles affects the SiNW etch rate in a non-monotonic manner and provides an additional mechanism to control the porosity of the resulting nanowires. Likewise, the etchant composition has a significant effect on the resulting nanostructures. There is an appropriate *χ* window within which uniform SiNW arrays can be formed. For H_2_O concentrations between 46 and 48 M, this *χ* range is 0.85–0.98; more dilute etchants increase the minimum *χ* due to sluggish etching at the SiNW base. The formation of Si nanostructures during Ag-catalyzed chemical etching is a result of the competing effects of different factors, namely, deposited Ag-catalyzed Si dissolution, re-nucleated Ag-catalyzed Si dissolution, hole diffusion, and reactant diffusion through etched pores, as reflected by the etching kinetics at the tip and base of the Si nanostructures. These factors result in a gradually decreasing etch rate over time, with re-nucleated Ag-catalyzed etching being dominant for low *χ* values and impeded diffusion being more pronounced for high *χ* values. These mechanisms can be exploited to control the porosity of SiNWs of the same length fabricated with different etch times. In particular, suppressing Ag re-nucleation and hole diffusion using higher *χ* values can effectively decrease pore density, while more controlled porosification can be achieved using dilute etchants owing to slower reactant diffusion and longer etch times. Since the general mechanism of Ag- and Au-catalyzed MACE of Si is similar [11, 33, 35], the findings of this study may also apply to porous SiNWs fabricated with Au metal catalyst, except that metal ion-induced etching is expected to be greatly suppressed and hole diffusion acts as the main mechanism of porosification and tip etching.

## Additional file

Additional file 1: Supplementary information. Details on etchant composition and etch durations used, method for determining the thickness of etched bulk Si, visual appearance of Si samples after etching, SEM images of SiNWs fabricated using high HF–H_2_O_2_ molar ratios, graphs of SiNW etch rates versus time, and additional TEM images of SiNWs showing varied porosity.

## Abbreviations

AgNP: Ag nanoparticle
DI: Deionized
MACE: Metal-assisted chemical etching
SEM: Scanning electron microscopy
SiNW: Silicon nanowire
TEM: Transmission electron microscopy
*χ*: HF–oxidant molar ratio
