# Supplementary material for: Unraveling the Morphological Evolution and Etching Kinetics of Porous Silicon Nanowires During Metal-Assisted Chemical Etching
Source: Nanoscale Res Lett. 2017 Jun 2;12:385. doi: 10.1186/s11671-017-2156-z (PMC5457386; doi:10.1186/s11671-017-2156-z)
Supplement: Additional file 1: — Supplementary information. Details on etchant composition and etch durations used, method for determining the thickness of etched bulk Si, visual appearance of Si samples after etching, SEM images of SiNWs fabricated using high HF–H2O2 molar ratios, graphs of SiNW etch rates versus time, and additional TEM images of SiNWs showing varied porosity. [file 11671_2017_2156_MOESM1_ESM.docx]

**Supplementary Information**

Unraveling the Morphological Evolution and Etching Kinetics of Porous Silicon Nanowires during Metal-Assisted Chemical Etching

Lester U. Vinzons^1^, Lei Shu^2,3^, SenPo Yip^2,3^, Chun-Yuen Wong^3,4^, Leanne L. H. Chan^1,5,*^, and Johnny C. Ho^2,3,5,6,*^

^1^Department of Electronic Engineering, City University of Hong Kong, Kowloon, Hong Kong

^2^Department of Physics and Materials Science, City University of Hong Kong, Kowloon, Hong Kong

^3^Shenzhen Research Institute, City University of Hong Kong, Shenzhen, 518057, P.R. China

^4^Department of Biology and Chemistry, City University of Hong Kong, Kowloon, Hong Kong

^5^Center for Biosystems, Neuroscience, and Nanotechnology, City University of Hong Kong, Kowloon, Hong Kong

^6^State Key Laboratory of Millimeter Waves, City University of Hong Kong, Kowloon, Hong Kong

*Correspondence: leanne.chan@cityu.edu.hk; johnnyho@cityu.edu.hk

**Table S1** Composition of HF–H_2_O_2_ Etchants for MACE of Si Nanostructures in the Study

| [H_2_O] (M) | *χ^a^* | [HF] (M) | [H_2_O_2_] (M) | 48% HF volume (ml) | 35% H_2_O_2_ volume (ml) | H_2_O  volume (ml) | Total volume (ml) |
| --- | --- | --- | --- | --- | --- | --- | --- |
| 46 | 0.7 | 7.06 | 3.03 | 3.840 | 3.904 | 7.256 | 15 |
| 46 | 0.75 | 7.75 | 2.58 | 4.216 | 3.334 | 7.451 | 15 |
| 46 | 0.8 | 8.48 | 2.12 | 4.610 | 2.734 | 7.655 | 15 |
| 46 | 0.85 | 9.24 | 1.63 | 5.026 | 2.104 | 7.870 | 15 |
| 46 | 0.9 | 10.05 | 1.12 | 5.463 | 1.440 | 8.097 | 15 |
| 46 | 0.95 | 10.90 | 0.57 | 5.924 | 0.740 | 8.336 | 15 |
| 46 | 0.99 | 11.61 | 0.12 | 6.312 | 0.151 | 8.537 | 15 |
| 48 | 0.7 | 5.58 | 2.39 | 3.032 | 3.083 | 8.885 | 15 |
| 48 | 0.75 | 6.12 | 2.04 | 3.329 | 2.633 | 9.038 | 15 |
| 48 | 0.8 | 6.70 | 1.67 | 3.641 | 2.159 | 9.200 | 15 |
| 48 | 0.85 | 7.30 | 1.29 | 3.969 | 1.661 | 9.370 | 15 |
| 48 | 0.9 | 7.94 | 0.88 | 4.314 | 1.137 | 9.549 | 15 |
| 48 | 0.92 | 8.20 | 0.71 | 4.458 | 0.920 | 9.623 | 15 |
| 48 | 0.95 | 8.61 | 0.45 | 4.678 | 0.584 | 9.737 | 15 |
| 48 | 0.98 | 9.03 | 0.18 | 4.907 | 0.238 | 9.856 | 15 |
| 48 | 0.99 | 9.17 | 0.09 | 4.985 | 0.119 | 9.896 | 15 |
| 50 | 0.7 | 4.09 | 1.75 | 2.225 | 2.262 | 10.513 | 15 |
| 50 | 0.75 | 4.49 | 1.50 | 2.443 | 1.931 | 10.626 | 15 |
| 50 | 0.8 | 4.91 | 1.23 | 2.671 | 1.584 | 10.745 | 15 |
| 50 | 0.85 | 5.36 | 0.95 | 2.912 | 1.219 | 10.869 | 15 |
| 50 | 0.9 | 5.82 | 0.65 | 3.165 | 0.834 | 11.000 | 15 |
| 50 | 0.95 | 6.31 | 0.33 | 3.433 | 0.429 | 11.139 | 15 |
| 50 | 0.99 | 6.73 | 0.07 | 3.657 | 0.088 | 11.255 | 15 |

^a^HF–H_2_O_2_ molar ratio, $\text{}\text{ }\text{=}\text{ }\frac{\text{[HF]}}{\text{[HF] +}\text{ }\text{[}\text{H}_{\text{2}}\text{O}_{\text{2}}\text{]}}$


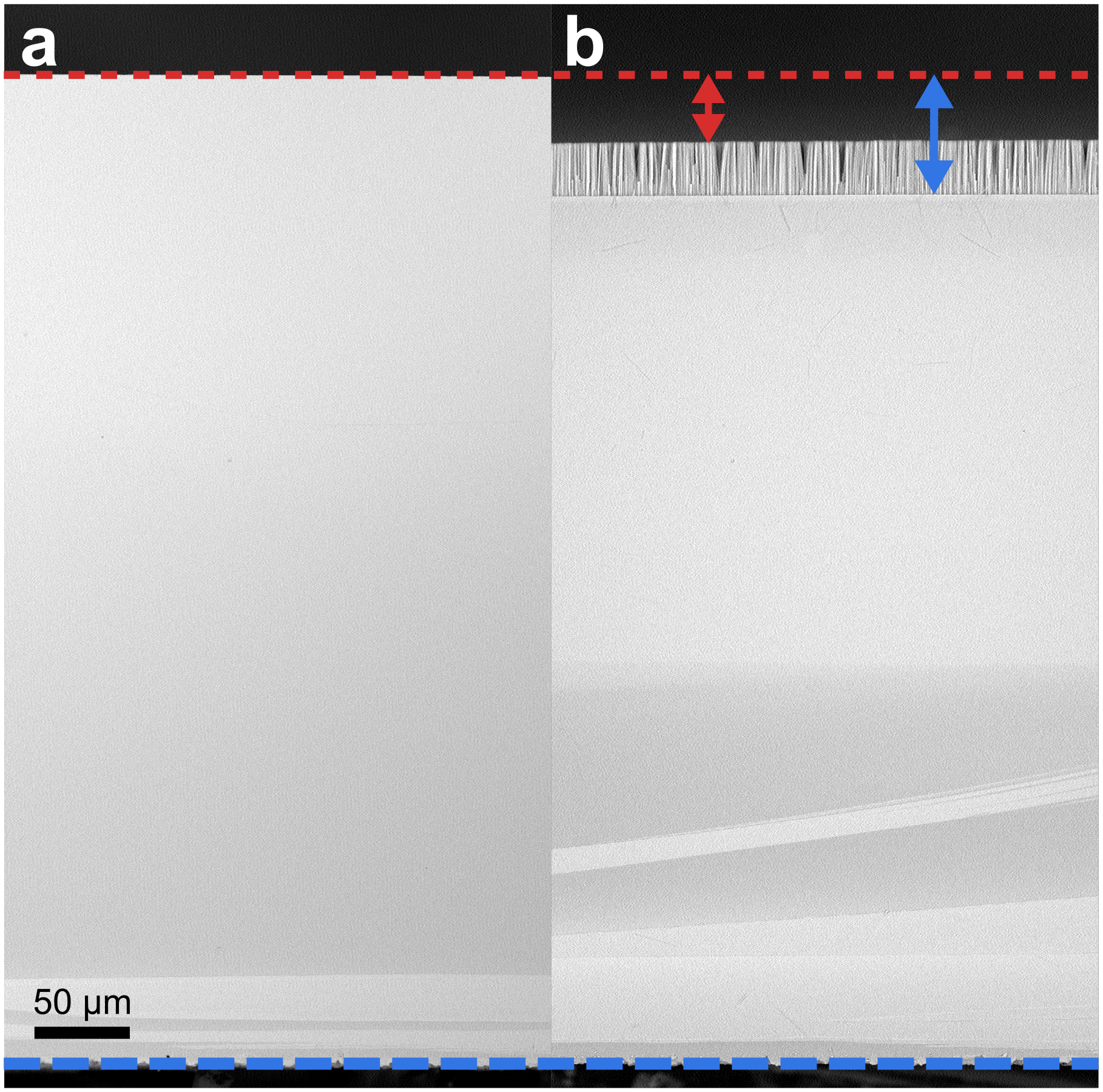


**Fig. S1** Determination of the etched bulk Si with respect to the tip and base of the Si nanostructures. Cross-sectional SEM images of **(a)** an unetched Si sample and **(b)** etched sample with SiNWs. The samples are aligned at the backside (marked with the blue dashed line), which was protected by photoresist during etching. The red dashed line shows the location of the top surface of the Si wafer before etching. The red and blue arrows show the thickness of the etched bulk Si with respect to the tip and base of the Si nanostructure, respectively.


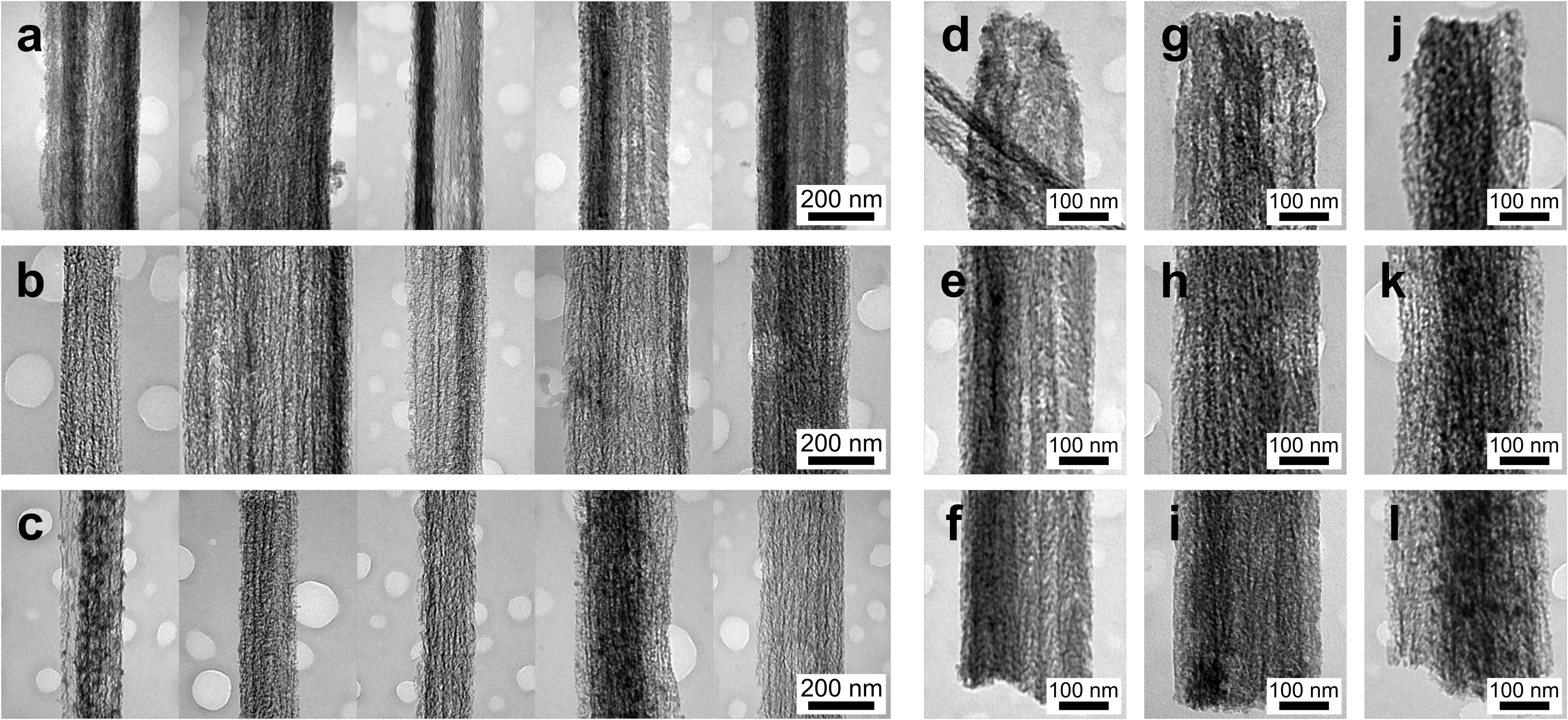


**Fig. S2** TEM images of SiNWs fabricated with electrolessly deposited Ag using different deposition times. **(a–c)** Middle section of randomly selected SiNWs from samples with Ag deposition times of **(a)** 4 min, **(b)** 10 min, and **(c)** 15 min. **(d–l)** Representative SiNWs from samples with Ag deposition times of **(d–f)** 4 min, **(g–i)** 10 min, and **(j–l)** 15 min showing the **(d, g, j)** top, **(e, h, k)** middle, and **(f, i, l)** bottom sections of the nanowires. Electroless deposition was performed in a solution composed of 0.005 M AgNO_3_ and 4.8 M HF, while etching was done in a solution containing 48 M H_2_O and 0.95 HF–H_2_O_2_ ratio.


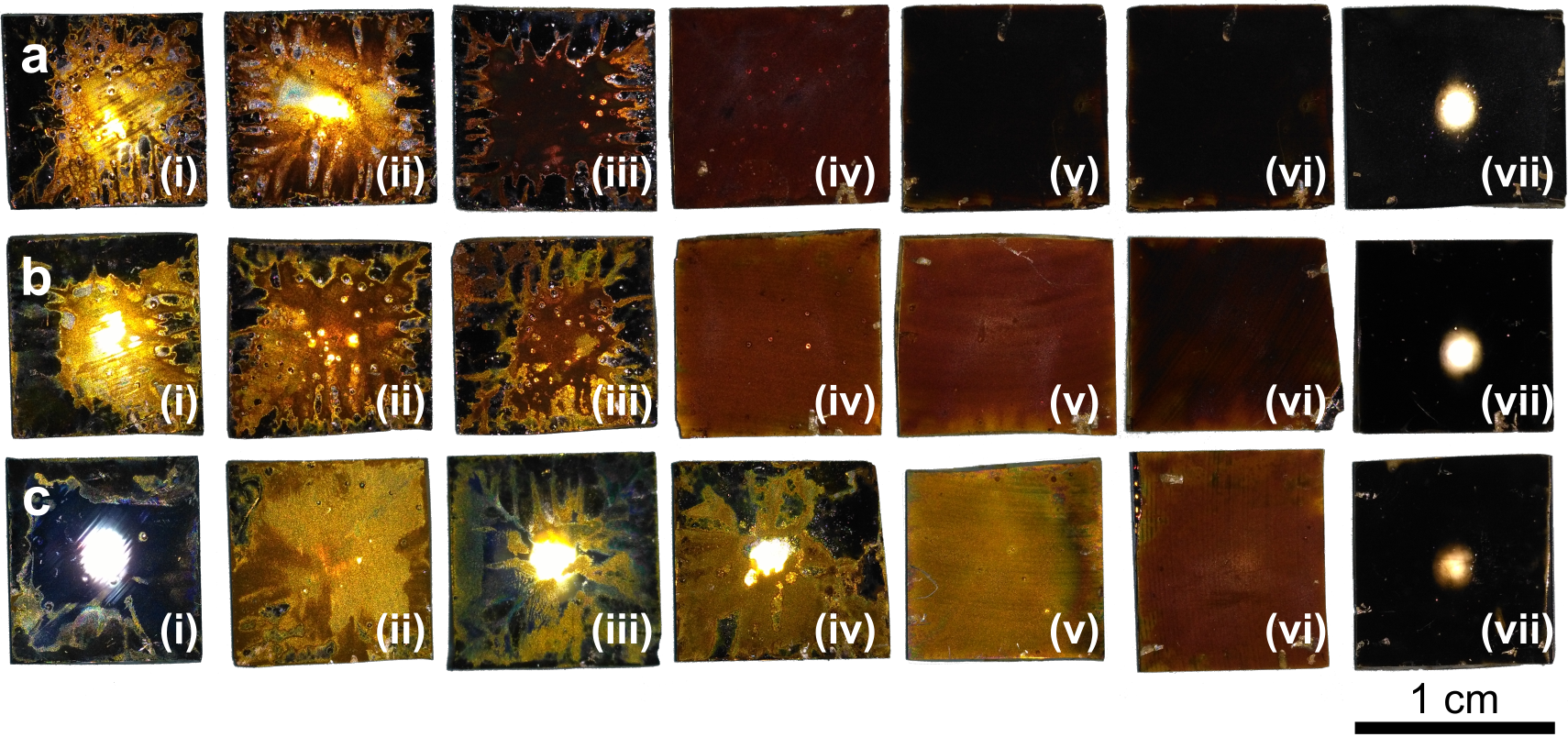


**Fig. S3** The 1 cm × 1 cm etched Si samples as seen with the naked eye after 30 min etching in HF–H_2_O_2_ solutions. The etchants had H_2_O concentrations of **(a)** 46 M, **(b)** 48 M, and **(c)** 50 M, and HF–H_2_O_2_ molar ratios of **(i)** 0.7, **(ii)** 0.75, **(iii)** 0.8, **(iv)** 0.85, **(v)** 0.9, **(vi)** 0.95, and **(vii)** 0.99. Lamp was shone at the center of the samples to show reflectivity.


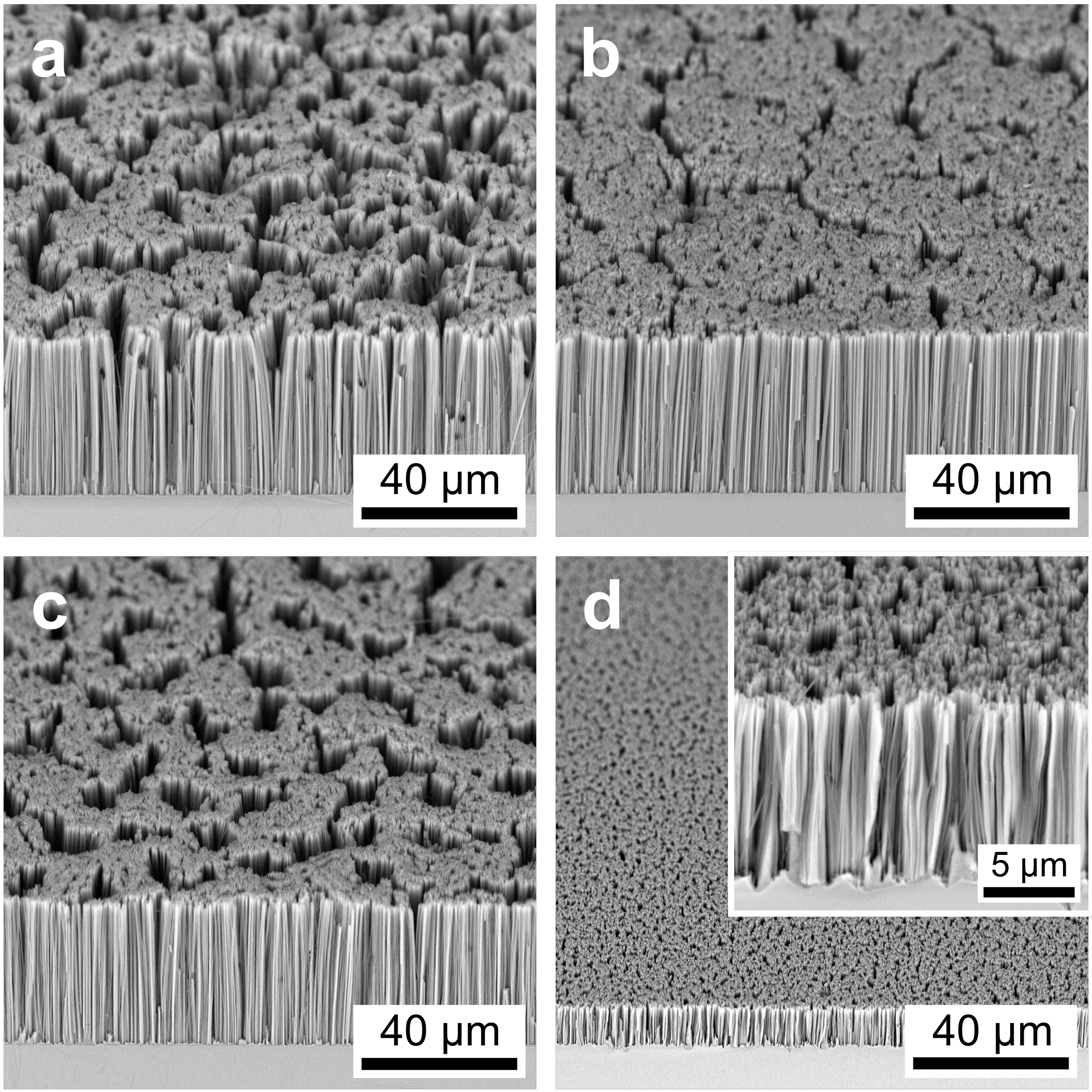


**Fig. S4** SEM images of SiNWs etched for 1 h in a solution composed of 48 M H_2_O and HF–H_2_O_2_ ratios of **(a, b)** 0.97, **(c)** 0.98, and **(d)** 0.99. Ag deposition was performed in a solution of 0.005 M AgNO_3_ and 4.8 M HF for **(a)** 4 min or **(b–d)** 10 min. The inset is an enlarged image of the corresponding sample.


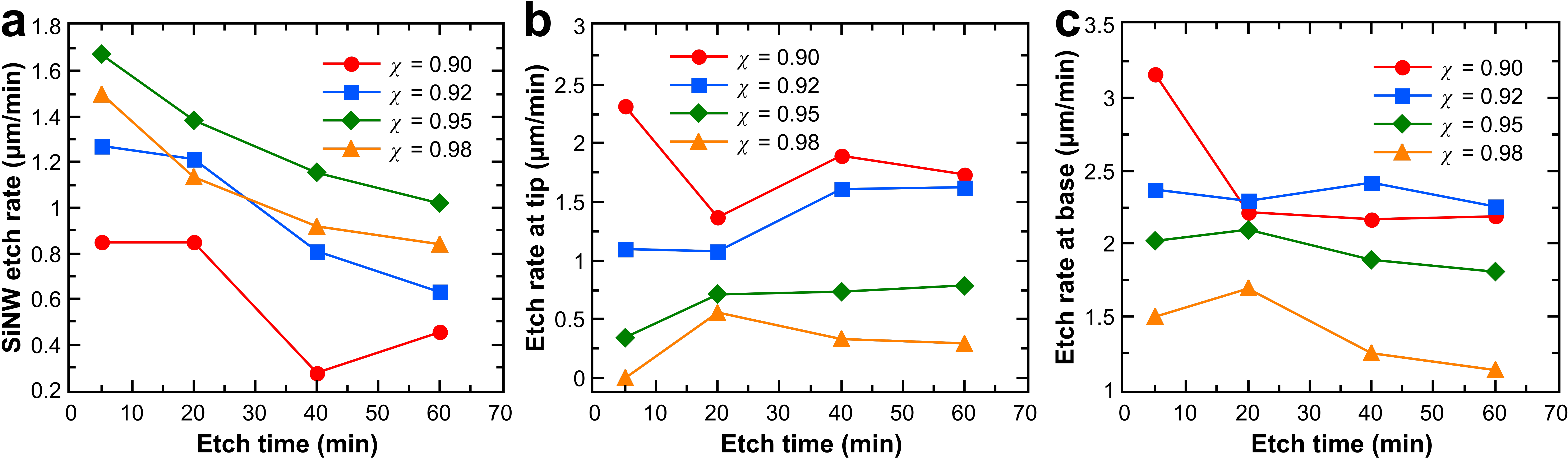


**Fig. S5** Variation of etch rates with time for different HF–H_2_O_2_ molar ratios at 48 M H_2_O. **(a)** Apparent etch rate of the SiNWs. **(b, c)** Etch rate of the bulk Si at the tip and base of the SiNWs, respectively.


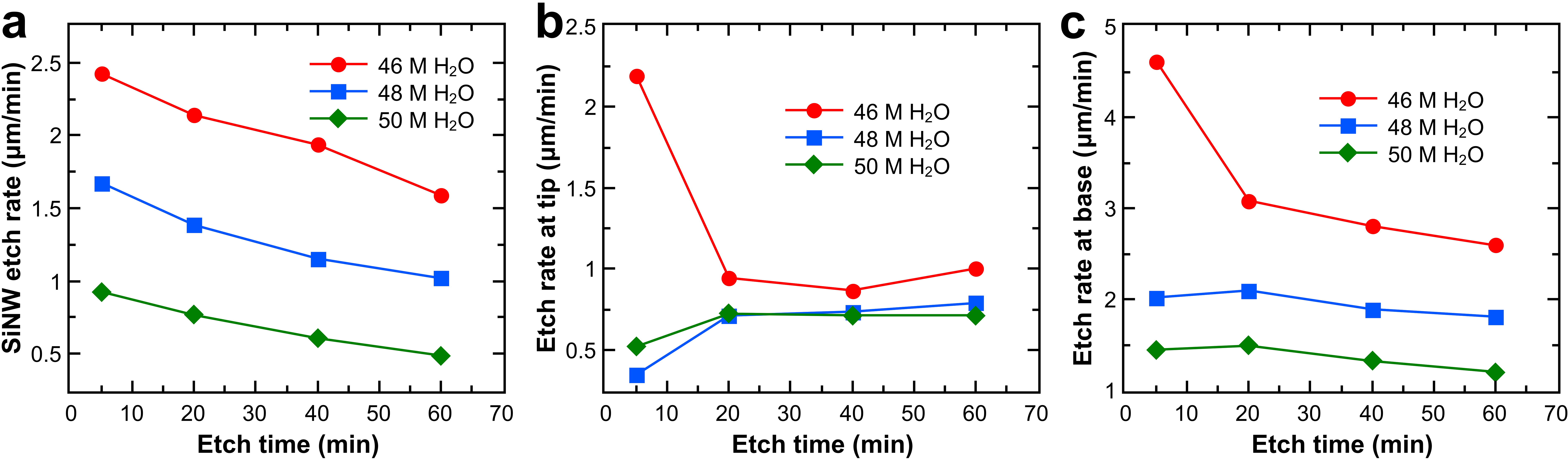


**Fig. S6** Variation of etch rates with time for different H_2_O concentrations at a fixed HF–H_2_O_2_ molar ratio of 0.95. **(a)** Apparent etch rate of the SiNWs. **(b, c)** Etch rate of the bulk Si at the tip and base of the SiNWs, respectively.

**Table S2** Etching Time for Each Etchant Composition to Obtain 20 μm Long Si Nanowires

| [H_2_O] (M) | HF–H_2_O_2_ molar ratio | etching time (min) | measured SiNW length (μm) |
| --- | --- | --- | --- |
| 48 | 0.92 | 15.03 | 19.760 |
| 48 | 0.95 | 14.20 | 21.257 |
| 48 | 0.98 | 18.17 | 22.455 |
| 46 | 0.95 | 8.47 | 21.557 |
| 50 | 0.95 | 29.02 | 21.856 |


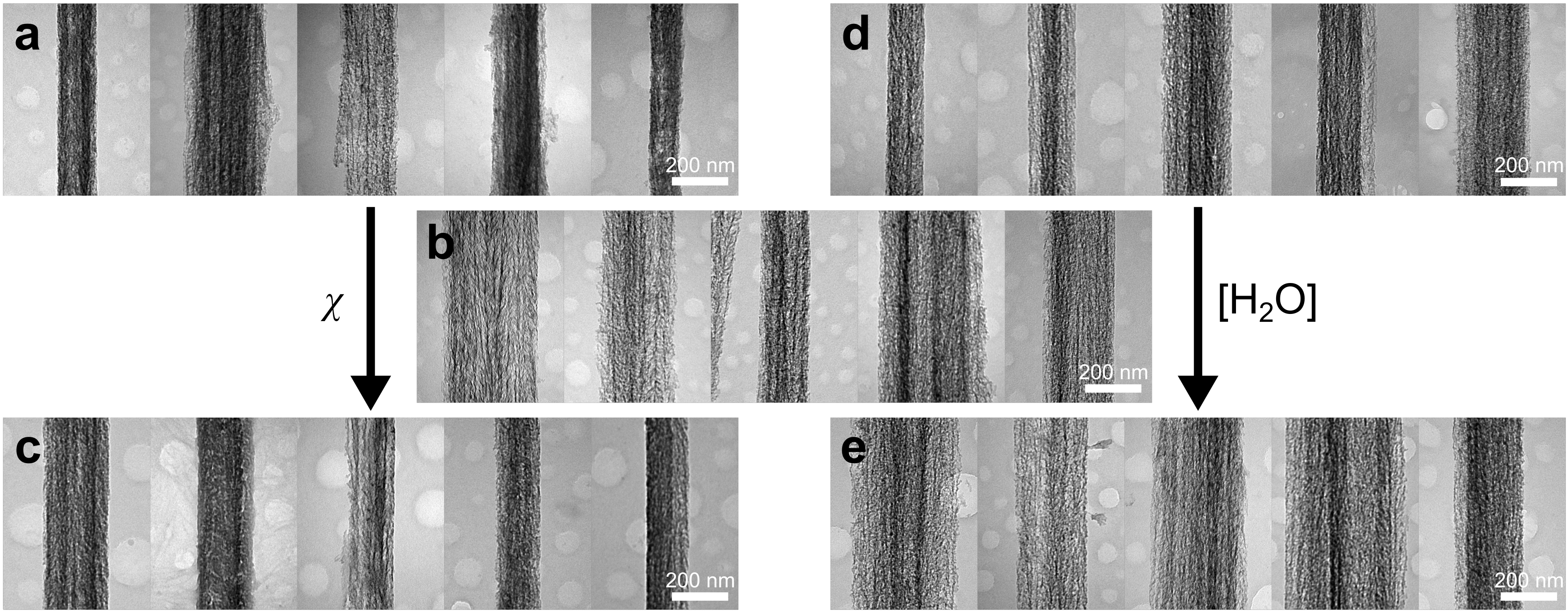


**Fig. S7** TEM images of five randomly selected SiNWs from samples etched in solutions containing different **(a–c)** HF–H_2_O_2_ molar ratios and **(d, b, e)** H_2_O concentrations. **(a–c)** HF–H_2_O_2_ molar ratio of the etchant was 0.92, 0.95, and 0.98, respectively, with [H_2_O] = 48 M. **(d, b, c)** H_2_O concentration of the etchant was 46 M, 48 M, and 50 M, respectively, with HF–H_2_O_2_ molar ratio = 0.95. SiNWs were fabricated with different time durations to achieve a length of ≈ 20 μm. All images show the middle section of the SiNWs.


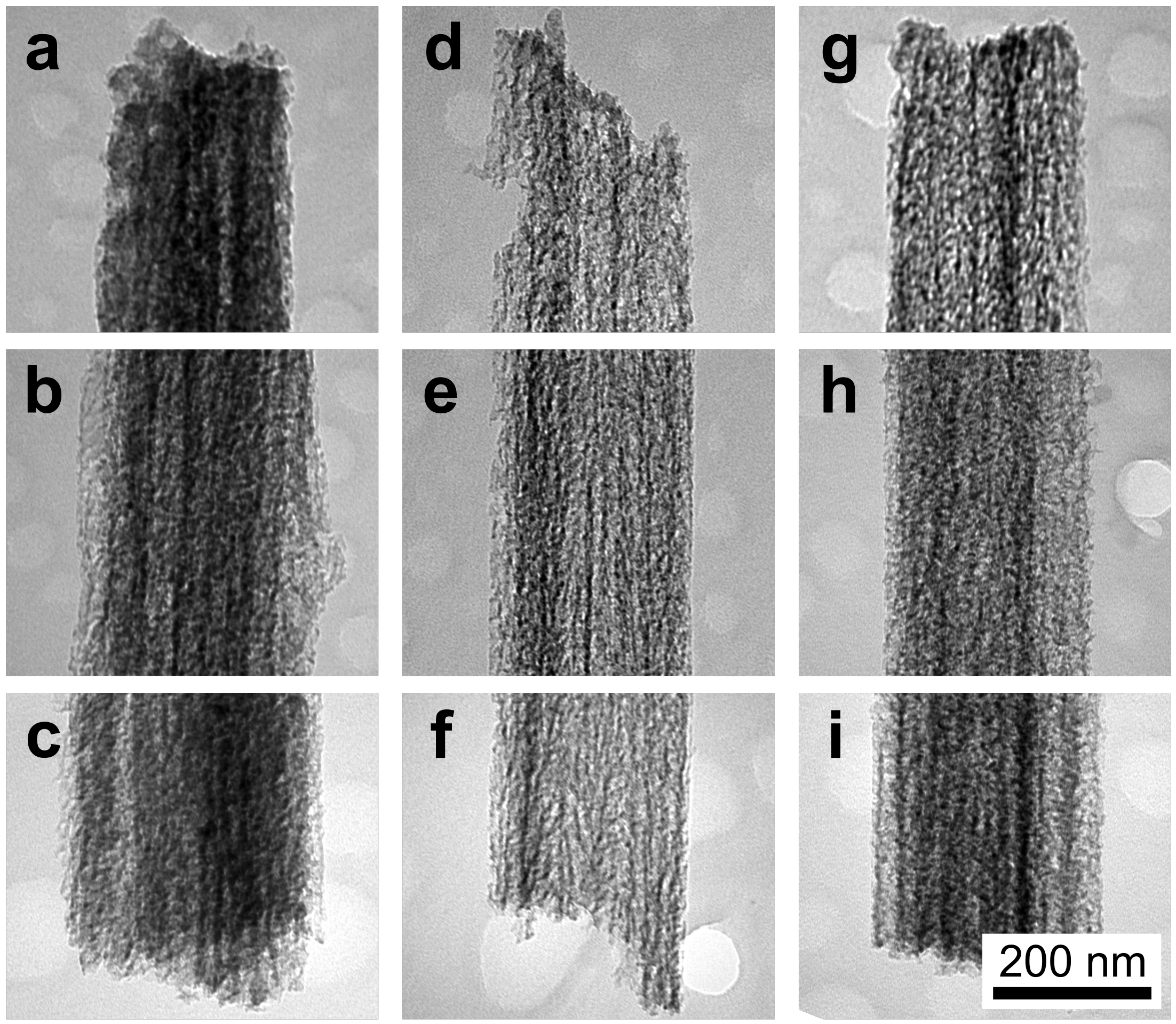


**Fig. S8** TEM images of representative SiNWs etched in a solution composed of **(a–c)** HF–H_2_O_2_ molar ratio *χ* = 0.92 and 48 M H_2_O, **(d–f)** *χ* = 0.95 and 48 M H_2_O, and **(g–i)** *χ* = 0.95 and 46 M H_2_O showing the **(a, d, g)** top, **(b, e, h)** middle, and **(c, f, i)** bottom sections of the nanowires. All SiNWs have a length of ≈20 μm. The scale bar applies to all images.
